# A Mouse Immunogenicity Model for the Evaluation of Meningococcal Conjugate Vaccines

**DOI:** 10.3389/fimmu.2022.814088

**Published:** 2022-01-20

**Authors:** Arun B. Arunachalam, Stacey Vile, Angel Rosas

**Affiliations:** ^1^ Analytical Sciences, R&D Sanofi Pasteur, Swiftwater, PA, United States; ^2^ Sanofi Medical Affairs, Sanofi Pasteur, Swiftwater, PA, United States

**Keywords:** animal, antibodies, bactericidal, IgG, mice, *Neisseria meningitides*, MenACYW-TT conjugate vaccine

## Abstract

The identification of an appropriate animal model for use in the development of meningococcal vaccines has been a challenge as humans are the only natural host for *Neisseria meningitidis*. Small animal models have been developed and are widely used to study the efficacy or immunogenicity of vaccine formulations generated against various diseases. Here, we describe the development and optimization of a mouse model for assessing the immunogenicity of candidate tetravalent meningococcal polysaccharide (MenACYW-TT) protein conjugate vaccines. Three inbred (BALB/c [H-2d], C3H/HeN [H-2k], or C57BL/6 [H-2b]) and one outbred (ICR [H-2g7]) mouse strains were assessed using serial two-fold dose dilutions (from 2 µg to 0.03125 µg per dose of polysaccharide for each serogroup) of candidate meningococcal conjugate vaccines. Groups of 10 mice received two doses of the candidate vaccine 14 days apart with serum samples obtained 14 days after the last dose for the evaluation of serogroup-specific anti-polysaccharide IgG by ELISA and bactericidal antibody by serum bactericidal assay (SBA). C3H/HeN and ICR mice had a more dose-dependent antibody response to all four serogroups than BALB/c and C57Bl/6 mice. In general, ICR mice had the greatest antibody dose-response range (both anti-polysaccharide IgG and bactericidal antibodies) to all four serogroups and were chosen as the model of choice. The 0.25 µg per serogroup dose was chosen as optimal since this was in the dynamic range of the serogroup-specific dose-response curves in most of the mouse strains evaluated. We demonstrate that the optimized mouse immunogenicity model is sufficiently sensitive to differentiate between conjugated polysaccharides, against unconjugated free polysaccharides and, to degradation of the vaccine formulations. Following optimization, this optimized mouse immunogenicity model has been used to assess the impact of different conjugation chemistries on immunogenicity, and to screen and stratify various candidate meningococcal conjugate vaccines to identify those with the most desirable profile to progress to clinical trials.

## Introduction

Invasive meningococcal disease (IMD), caused by *Neisseria meningitidis*, can rapidly progress to serious life-threatening disease in otherwise healthy individuals, typically characterized as meningitis and sepsis, with fatality rates up to 20% despite appropriate treatment (1). Six meningococcal serogroups (A, B, C, W, X and Y) are responsible for nearly all IMD cases worldwide, with regional variation as to the predominant causative serogroup (1–3). Reported rates of IMD incidence range considerably between countries with up to >200 cases per 100,000 people reported per year pre-vaccine introduction, with the highest burden predominantly in Africa (2, 3). Vaccination has considerably reduced the incidence of IMD worldwide (2).

Meningococcal polysaccharide vaccines that protect against one or more serogroups have been available for more than 40 years (1). Bacterial polysaccharides are considered to be T-cell independent antigens, most of which are poor immunogens when used as vaccines and can cause hypo-responsiveness in individuals (4). Although these polysaccharide antigens are immunogenic in adults, by crosslinking of B-cell receptors through their multivalent epitopes, they cannot induce immune memory. To circumvent this inherent problem, polysaccharides are conjugated to carrier proteins with T-cell epitopes, which convert them to T-cell dependent antigens. Such polysaccharide-protein conjugates can elicit robust immune responses that protect recipients from *Haemophilus influenzae* type b, *Neisseria meningitidis* and *Streptococcal pneumoniae* infections (5, 6).

Several factors can influence the immunogenicity of polysaccharide-protein conjugate vaccines; these include: the carrier protein, polysaccharide to protein ratio, O-acetylation of polysaccharide antigens, and the nature and the length of the spacer between the polysaccharide antigen and the carrier protein (5). A pre-clinical animal model is necessary during the early stage of vaccine development to evaluate and understand the potential influence of each factor alone, and in combination, on immunogenicity. An optimized pre-clinical model can considerably reduce vaccine development duration and costs as it allows for expedited screening and selection of candidates with the most desirable profile for assessment in clinical trials. Animal models have been used successfully to elucidate the nature of the immune response specific to the carrier protein in glycoprotein conjugate vaccines. For example, conjugate carriers tetanus toxoid (TT) and diphtheria toxoid (DT), but not cross-reacting material 197 (CRM_197_), induced antibody responses that protected mice and guinea pigs against lethal challenge with the respective toxins (7). Although animal models have helped predict how well some vaccines work in humans, careful consideration as to their inherent limitations should allow the identification of an ‘optimal’ model that more accurately predicts immunogenicity outcomes in clinical trials (8).

Here, we describe the development and optimization of a mouse immunogenicity model for the evaluation of candidate meningococcal polysaccharide-protein conjugate vaccines.

## Methods

### Ethical Statement

Animal studies described here were approved by the Institutional Animal Care and Use Committee (Sanofi Pasteur, Swiftwater, PA) and undertaken in accordance with the recommendations in ‘The Guide for the Care and Use of Laboratory Animals’ published by the National Research Council (Washington DC).

### Immunogen

*Neisseria meningitidis* polysaccharides specific to serogroups A, C, W or Y were derivatized and conjugated to a carrier protein, either TT or DT (9). These monovalent polysaccharide-protein conjugate vaccine preparations were blended to form bulk tetravalent formulations (MenACYW-TT or MenACYW-DT). MenACYW-TT vaccine formulation was two-fold serially diluted in 0.85% saline from 2 µg/dose down to 0.03125 µg/dose of each meningococcal serogroup polysaccharide. These serial dilutions were prepared on the day of immunization and used immediately. A non-conjugated (free polysaccharide) MenACYW preparation was also included for comparison at 5 µg (per serogroup) dose. MenACYW-DT stored under forced degradation conditions (56°C for 1 week) was also evaluated in the optimized mouse model to assess the impact on immune response of the candidate vaccine having undergone a short-term increase of storage temperature outside recommended storage conditions (2–8°C).

### Mouse Strains

Female inbred and outbred mouse strains (inbred strains BALB/c [H-2d], C3H/HeN [H-2k], or C57BL/6 [H-2b], and outbred strains ICR [H-2g7] or Swiss Webster [H-2q2]) aged 6–8 weeks were purchased from Hilltop Lab Animals Inc (Scottdale, Pennsylvania) and housed in groups of 10 mice/cage. The animals were rested for a minimum of three days prior to inclusion in the studies. Food and water were available *ad libitum*.

### Immunization

**Figure 1:**
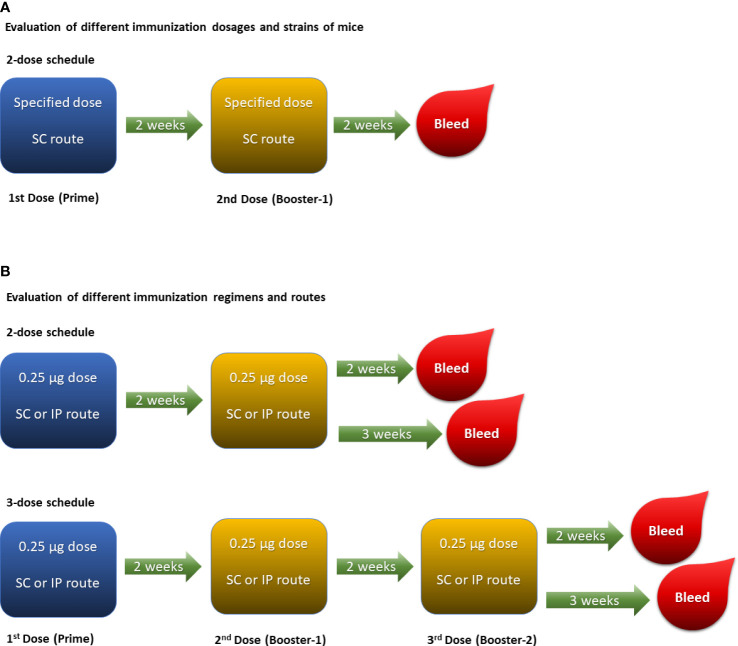
Experimental designs: **(A)**. Two-dose schedule given subcutaneously was performed to evaluate varying doses ranging from 2 µg/dose down to 0.03125 µg/dose of each meningococcal serogroup polysaccharide in different strains of mice. **(B)**. Two-dose and three-dose schedules were performed in ICR mice with 0.25 µg/dose of each meningococcal serogroup polysaccharide to evaluate different routes of immunization (i.e., SC and IP).

A number of parameters such as mouse strain, immunization dose, schedule and route were evaluated to develop an optimal immunogenicity model for assessing MenACYW conjugate vaccines. Initially, groups of 10 mice (four mouse strains assessed, excluding the Swiss Webster [H-2q2] strain due to supply issues) were injected subcutaneously with 0.25 mL of each of the candidate conjugate vaccine dose preparation or 0.85% saline as a control on days 0 and 14 (two-dose schedule; **Figure 1A**) to identify the optimal mouse strain and immunization dose. An ideal pre-clinical animal model should be sensitive enough to detect a drop in the potency of vaccines either due to alteration of conjugation parameters or deterioration. A three-dose immunization schedule was also assessed, in parallel to the two-dose immunization schedule, using the optimal mouse strain and dose identified, with the MenACYW-TT conjugate vaccine administered as three subcutaneous (SC) or intraperitoneal (IP) injections (on days 0, 14 and 28) (**Figure 1B**). Sera were collected two or three weeks after the last dose for assessment of total IgG and bactericidal antibody responses. Specific details on the immunization regimen of each study are provided in the results section.

The optimal mouse model was also used to assess its sensitivity to deterioration of MenACYW-DT. In this assessment, groups of 10 mice received SC injections on days 0 and 14 (two-dose schedule) of 0.25 µg per serogroup MenACYW-DT previously stored under forced degradation conditions (56°C for 1 week) or stored under recommended conditions (2–8°C). Serum samples were obtained two weeks after the last dose (on day 28) for assessment of total IgG.

In a separate assessment, groups of 10 Swiss Webster (H-2q2) mice were administered SC doses of 5 µg per serogroup MenACYW free polysaccharide or 0.25 µg per serogroup MenACYW-DT on days 0 and 14 (two-dose schedule), with serum samples obtained two weeks after the last dose (on day 28) for assessment of total IgG.

### Serological Assessments

The serogroup-specific anti-polysaccharide (A, C, W and Y) IgG titers from immunized mice were determined by ELISA. In brief, the wells of polystyrene 96-well microtiter plates (Immulon II flat bottom, ThermoFisher Scientific [Thermo LabSystems]) were coated with meningococcal polysaccharide antigens complexed with methylated human serum albumin (100 µL of solution/well containing 5 µg/mL of each serogroup) for a minimum of 15 hours at 2–8°C. The antigen solution was then decanted and the wells blocked with 5% fetal bovine serum in 0.01M phosphate-buffered saline (PBS) containing 0.1% Brij-35 (30% w/v solution). Sera from individual mice as well as working reference standards and control sera (100 μL of each sample) were two-fold serially diluted in diluent buffer (5% fetal bovine serum in 0.01M PBS containing 0.1% Brij-35 [30% w/v solution]), added to the antigen-coated wells, and the plates incubated for 90–120 minutes at 37°C. The plates were washed five times with 350 µl of wash buffer per well using an automated microplate washer (e.g., Dynatech Ultrawash Plus [Dynatech Laboratories]), and the well filled with peroxidase-conjugated goat anti-mouse IgG (γ) (Kirkegaard & Perry Laboratories Inc, Gaithersburg, MD). The plates were washed again five times with 350 µL of wash buffer per well using the automated microplate washer, before the addition of 100 µL of TMB (3,3’5,5’-Tetramethylbenzidine) and incubation for 15 minutes at room temperature. The colorimetric reaction was stopped by adding 50 µL of 1M phosphoric acid. The plates were read using a Molecular Devices plate reader with automix at 450 nm, with the reference wavelength at 650 nm. The concentration (in mouse ELISA units [MEU] or µg/mL) of the serogroup-specific anti-polysaccharide IgG antibodies in sera was derived using Softmax^®^ Pro, by extrapolation from a standard reference curve. For the generation of reference standard serum, female ICR mice (6 to 8 weeks) were injected subcutaneously with a MenACYW-TT conjugate formulation at 1 µg of polysaccharide per serogroup in 0.25 mL dose on days 0 and 14, and serum samples were obtained two weeks after the last dose (on day 28). Individual sera were pre-screened by ELISA and the sera that had detectable level of serogroup A specific antibodies were pooled. The pooled serum (reference standard) was qualified by four analysts over the period of 3 weeks that generated 70 independent IgG concentration values to each of the four meningococcal serogroup polysaccharides. Arithmetic mean for the data that followed a normal distribution and geometric mean for the data that did not follow a normal distribution by the Shapiro-Wilk normality test were calculated and assigned to the reference standard serum as serogroup specific IgG concentration values. Serogroup specific antibody concentrations, in the reference standard serum, expressed as Mouse ELISA Units (MEU) were derived from the serum dilutions that generated EC_50_ (i.e., half maximal effective concentration) of the sigmoidal response curve in the ELISA. Serogroup specific antibody concentrations, in the reference standard serum, expressed in µg/mL were calculated using purified serogroup specific mouse IgG antibodies.

Functional antibody responses in immunized mice were determined using an adapted SBA developed for human sera (10). In brief, the SBA was performed with meningococcal reference strains serogroup A (F8238), C (C-11), W (2515), and Y (3021) obtained from the Centers for Disease Control and Prevention, Atlanta, Georgia. SBA testing of individual sera could not be performed due to limited volumes; sub-pools in a given test group were prepared by combining two mouse sera to obtain five sub-pools per group. All sera were heat-treated at 56°C for 30 minutes before assessment. Sera from control mice were pooled and used as a negative control. Sera containing high serogroup-specific anti-polysaccharide IgG titers obtained from vaccinated mice were pooled and used as positive controls. The meningococcal serogroups strains in their log phase of growth were suspended in bactericidal buffer (Dulbecco’s PBS plus 0.1% dextrose) to obtain an optimal bacterial concentration (until an absorbance of 0.200 ± 0.050 at 600 nm is obtained). For the SBA, two-fold dilutions of test sera were added to the wells of 96-well, flat bottom sterile microtiter plates (Corning Costar Catalog #3997) containing the meningococcal serogroup strains and rabbit serum complement. After initial incubation for 60–90 minutes at 37°C in 5% CO_2_ atmosphere, an agar overlay medium was added, allowed to harden and incubated overnight at 37°C in 5% CO_2_ atmosphere. For the agar overlay medium, SeaKem ME agarose (Lonza) containing Trypticase Soy broth (Becton Dickinson) was melted in an autoclave at 121°C for 20 minutes and then equilibrated to 50°C ± 1°C for at least 30 minutes in a water bath prior to use. After all these steps each sample well contained 50 µL of diluted serum sample, 12.5 µL of bacterial suspension at an optimal concentration, 12.5 µL of rabbit complement serum and 100 µL of equilibrated agar overlay. Bacterial colonies were counted after overnight incubation, and the endpoint titer determined by the reciprocal serum dilution yielding ≥50% killing compared with the mean of the complement control wells.

### Statistical Analysis

Descriptive statistics were reported for all immunogenicity variables; serogroup-specific anti-polysaccharide IgG and bactericidal antibody responses were described using geometric mean concentrations (GMC) and geometric mean titers (GMT), respectively.

## Results

Unless a specific mouse strain is indicated, the results are presented for ICR mice. The experimental design is summarized in **Figure 1**. Control groups of mice were immunized with 0.85% physiological saline used to dilute the formulations, utilizing the same schedule as that of the immunized groups. Saline control groups, irrespective of mouse strain used, were consistently below the lower limit of detection of the IgG enzyme-linked immunosorbent assay (ELISA) and serum bactericidal antibody assay (SBA). Data for the control groups are not shown.

### Mouse Strain and Immunization Dose

**Figure 2:**
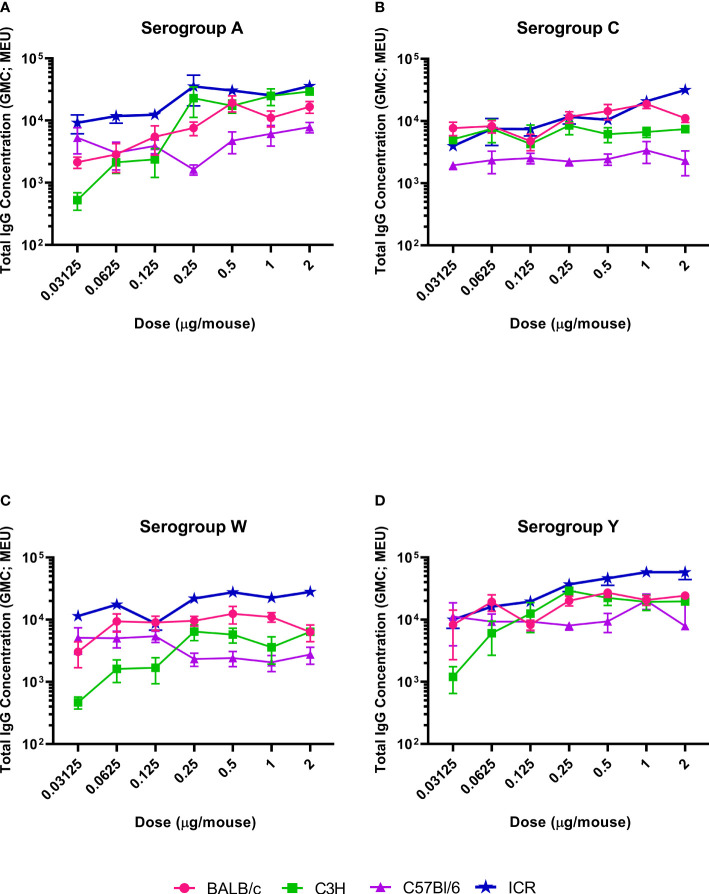
Serogroup-specific anti-polysaccharide IgG dose responses. Mice were injected subcutaneously with MenACYW-TT conjugated vaccine on days 0 and 14 (two-dose schedule). Serogroup-specific anti-polysaccharide IgG antibodies were measured by ELISA in serum obtained two weeks after the last dose (day 28). Error bars represent standard error for each group. The antibody responses obtained for serogroups A, C, W and Y are shown in **(A–D)** respectively. The dose response curves obtained for different strains of mice are shown in different colors as indicated in the figure labels.

**Figure 3:**
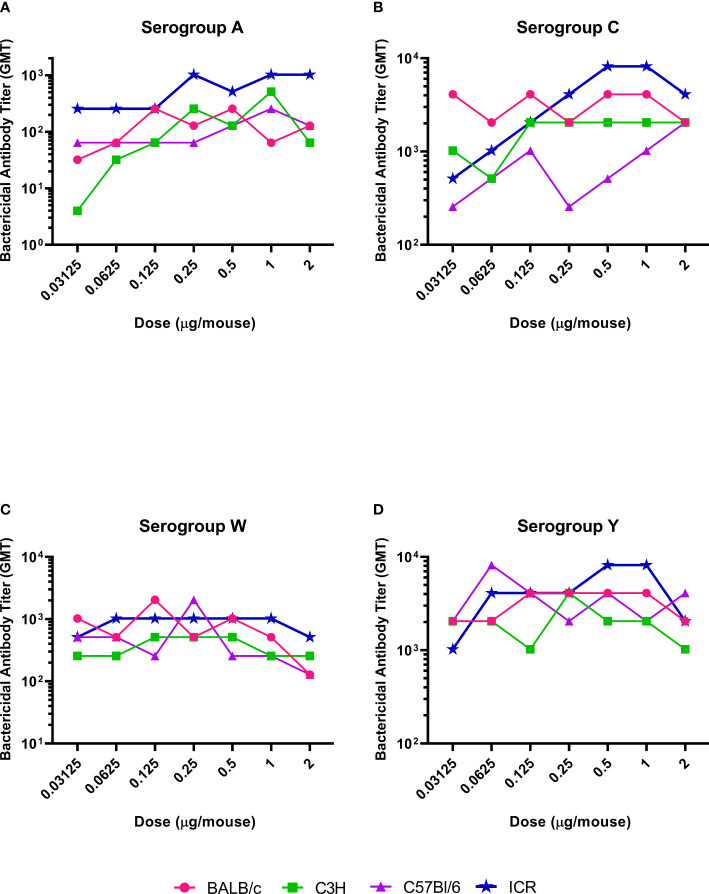
Serogroup-specific bactericidal antibody dose responses. Mice were injected subcutaneously with MenACYW-TT conjugated vaccine on days 0 and 14 (two-dose schedule). Bactericidal antibodies were measured by serum bactericidal assay in the presence of rabbit complement using serum obtained two weeks after the last dose (day 28). The antibody responses obtained for serogroups A, C, W and Y are shown in **(A–D)** respectively. The dose response curves obtained for different strains of mice are shown in different colors as indicated in the figure labels.

The serogroup-specific anti-polysaccharide IgG and bactericidal antibody responses following a two-dose SC immunization schedule with the tetravalent meningococcal serogroups A, C, Y, and W conjugated to TT (MenACYW-TT) vaccine are summarized by mouse strain in **Figures 2** and **3**, respectively. All four mouse strains elicited antibody responses especially for the higher doses of MenACYW-TT conjugate vaccine. In general, antibody responses (total IgG and bactericidal) increased with increasing dose across all mouse strains, but the responses tended to be highest in ICR mice and lowest in C57BL/6 mice. Overall, the dose-response curves obtained were steeper for ICR and C3H mice than for BALB/c and C57Bl/6 mice. C3H mice had a consistent change in the antibody responses between doses. However, the antibody responses to the four serogroups were not as robust as in ICR mice. Total IgG responses, induced by the 0.25 µg dose, were toward the center of the ICR dose response curves, except those for serogroup A which neared saturation. Additionally, bactericidal antibody responses were highest overall and showed a dose response to serogroups A, C and Y in ICR mice. Therefore, the 0.25 µg of each serogroup polysaccharide per dose and ICR mice were selected for subsequent studies evaluating the immunogenicity of MenACYW-TT or MenACYW-DT vaccine formulations. Similar conclusions were reached when tetravalent polysaccharide-protein conjugate formulations using different carrier proteins were assessed (data not shown).

### Immunization Regimen and Route

**Figure 4:**
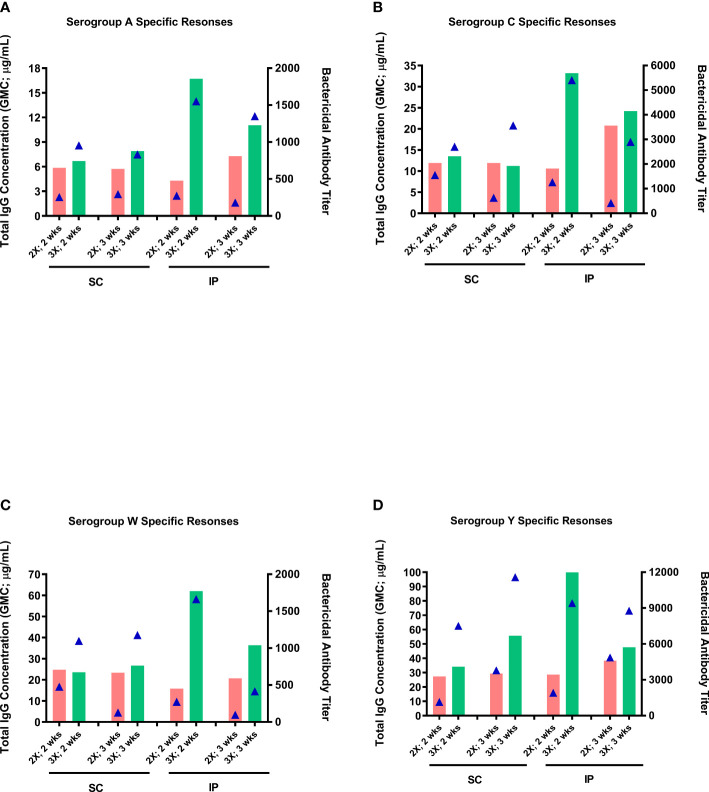
Effect of administration route and number of immunization doses on immunogenicity of the MenACYW-TT conjugated vaccine. Groups of 10 ICR mice received subcutaneous (SC) or intraperitoneal (IP) injections with of 0.25 µg per serogroup MenACYW-TT conjugated vaccine as two-dose (days 0 and 14; 2X) or three-dose (on days 0, 14 and 28; 3X) schedules. Serum samples were obtained two weeks (2 wks) and three weeks (3 wks) after the last dose for assessment of total IgG (bars) and bactericidal (triangles) antibody responses. The antibody responses obtained for serogroups A, C, W and Y are shown in **(A–D)** respectively.

The effect of the number of doses (two-dose vs three-dose schedule) and route of administration (SC vs IP) on antibody responses was assessed using the optimal dose (0.25 µg of each serogroup polysaccharide per dose) and mouse strain (ICR mice) identified in the dose-ranging studies. Overall, higher antibody responses (markedly more so with bactericidal antibody responses) were induced with the three-dose schedule than the two-dose schedule, irrespective of immunization route (**Figure 4**). Although antibody responses following SC and IP administration were similar with the two-dose schedule, IP administration tended, in most cases, to induce considerably higher levels of antibodies than with SC immunization with the three-dose schedule. In general, the antibody responses either stayed the same or were noticeably decreased when the sera were collected three weeks after the last dose compared to two weeks after.

### Free Versus Conjugated Meningococcal Polysaccharides

**Figure 5:**
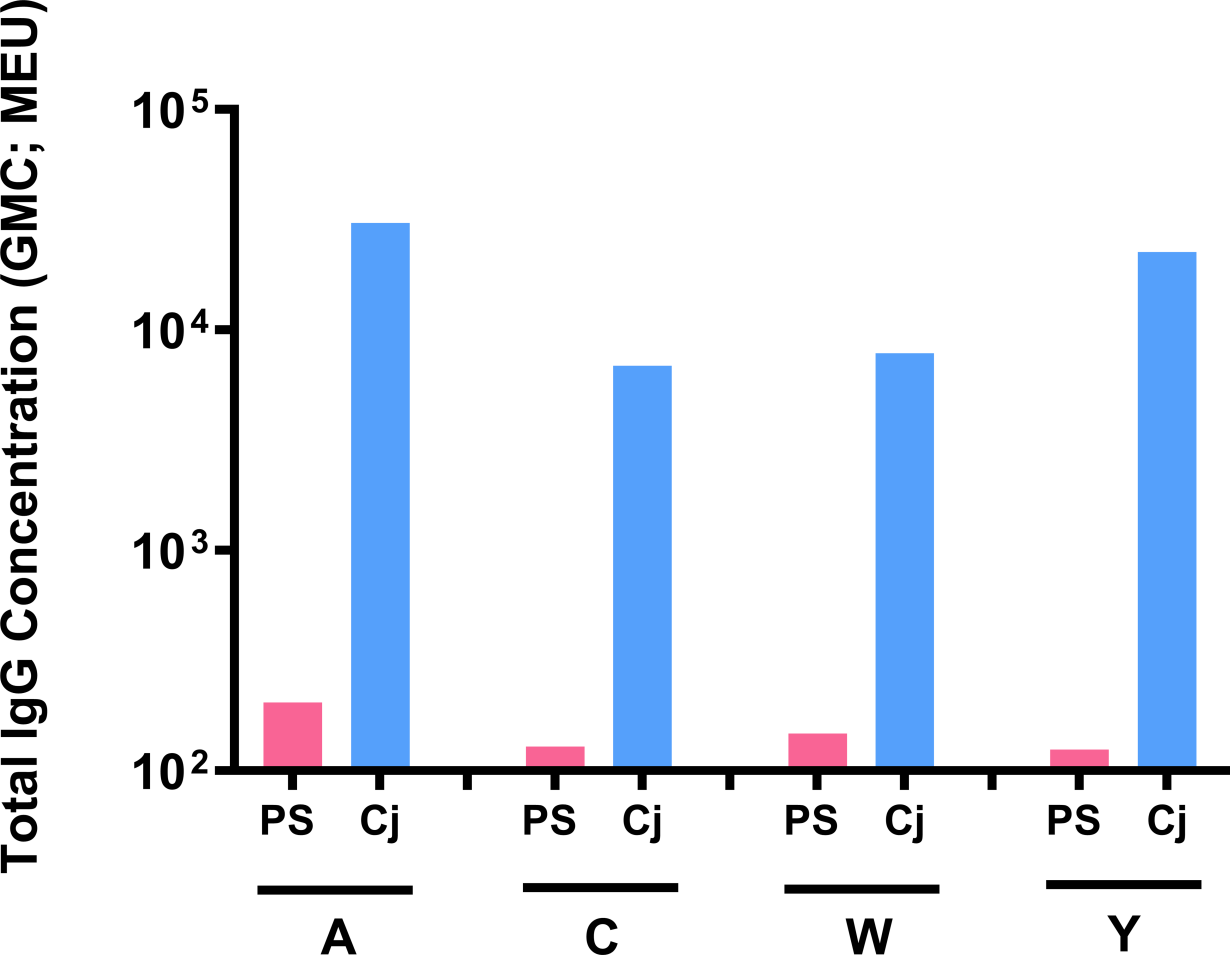
Antibody response following immunization with MenACYW (free polysaccharide) and MenACYW-DT (conjugated polysaccharides). Groups of 10 Swiss Webster (H-2q2) mice were administered subcutaneous (SC) doses of 5 µg per serogroup MenACYW polysaccharides or 0.25 µg per serogroup MenACYW-DT conjugated vaccine on days 0 and 14 (two-dose schedule), with serum samples obtained two weeks after the last dose (on day 28) for assessment of total IgG.

Outbred Swiss Webster mice were used to compare serogroup-specific anti-polysaccharide IgG responses following immunization with MenACYW free polysaccharide and the tetravalent meningococcal serogroups A, C, W and Y conjugated to DT (MenACYW-DT) conjugate vaccine; this mouse strain elicited antibody responses to MenACYW-DT similar to outbred ICR mouse strain (data not shown). Immunization with MenACYW-DT induced significantly higher total IgG levels than MenACYW free polysaccharide, even with a 20-times higher dose than the conjugated vaccine (**Figure 5**). MenACYW free polysaccharide induced negligible levels of serogroup-specific anti-polysaccharide IgG antibodies.

Isotyping profile showed that antibodies, although minimally induced by unconjugated free polysaccharide, were primarily IgM whereas antibodies induced by MenACYW-DT switched to IgG isotype (data not shown).

### Discrimination of Deteriorated Vaccines

**Figure 6:**
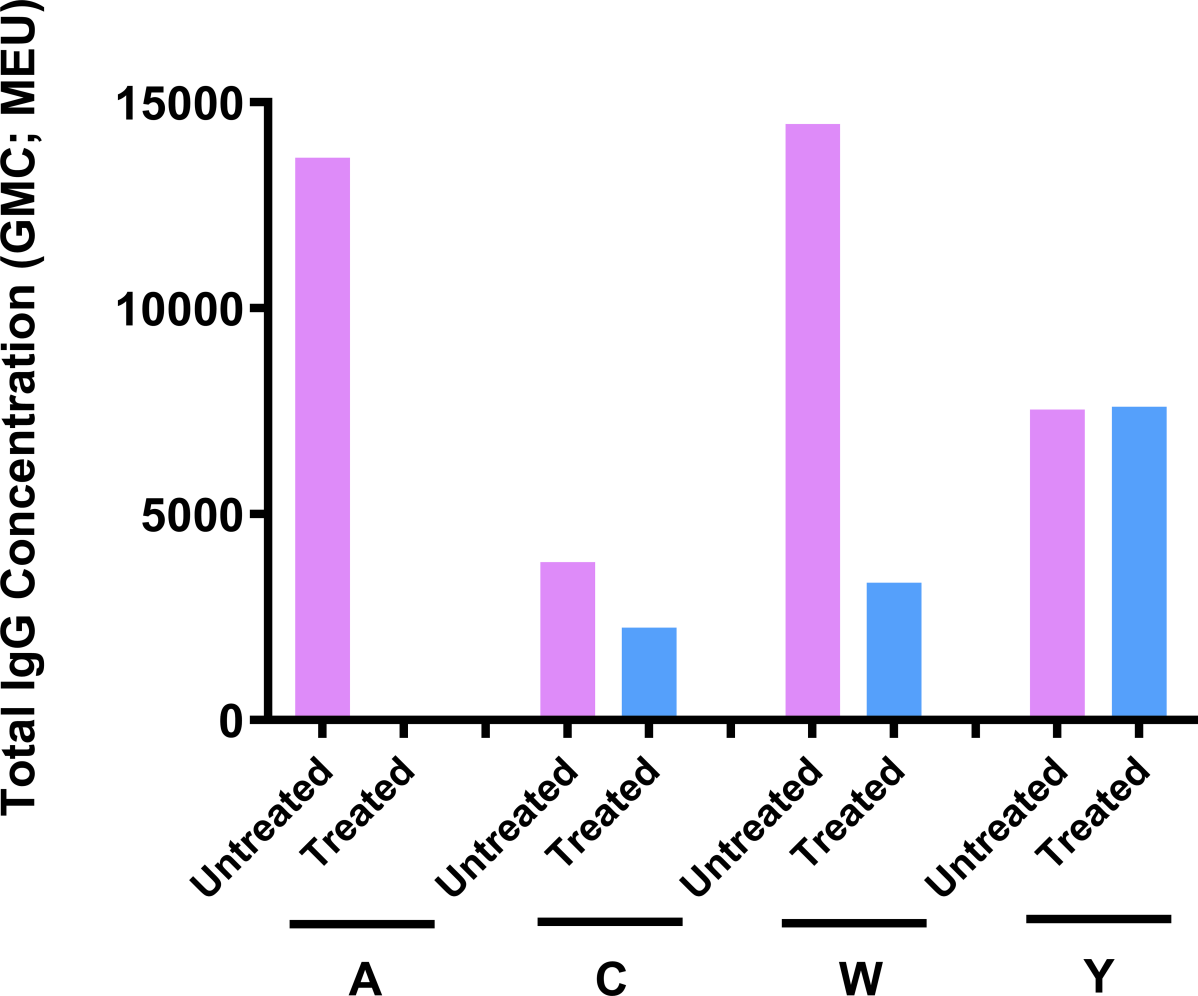
Sensitivity of the ICR mouse model to MenACYW-DT deterioration. Immunogenicity of MenACYW-DT stored under accelerated deterioration conditions (56°C for 1 week) was compared to that stored under recommended conditions (2–8°C). Groups of 10 ICR mice were injected with two subcutaneous (SC) doses on days 0 and 14 (two-dose schedule) of 0.25 µg per serogroup MenACYW-DT previously stored under accelerated deterioration (treated) or recommended (untreated) conditions. Serum samples were obtained two weeks after the last dose (on day 28) for assessment of total IgG.

The optimized mouse immunogenicity model (ICR mice, two-dose schedule, and 0.25 µg of each serogroup polysaccharide per dose) was also used to assess its sensitivity to deterioration of MenACYW-DT. Total IgG responses decreased significantly for serogroup A and W following storage of MenACYW-DT under accelerated deterioration conditions, and was moderately reduced for serogroup C but remained unaffected for serogroup Y (**Figure 6**). These results clearly demonstrate the sensitivity of the optimized mouse immunogenicity model to detect deterioration in the conjugated vaccine.

## Discussion

An animal model that recapitulates pathophysiology of a pathogen similar to that in humans would be ideal to predict vaccine efficacy. However, it is rare to find such an animal model and moreover, some challenge models depend on large animals which are cost-prohibitive and not suited for routine screening of vaccine candidates (11, 12). Therefore, many researchers rely on suitable biomarkers or correlates of protection as readouts to predict vaccine efficacy (8). Here, we selected both total IgG and protective (i.e., bactericidal) antibody responses in mice as biomarkers to identify the best meningococcal vaccine candidate(s) for the clinical studies. Our initial studies comparing mice, rats, and rabbits suggested that mice were the best species for assessing immunogenicity of MenACYW conjugate vaccines considering the antibody (total IgG and bactericidal) responses to all four serogroups as well as the cost effectiveness for including an adequate number of animals per group for statistical analysis (data not shown). Also, we found in these preliminary studies that female mice generated more robust antibody responses, and they were easier to handle than male mice (data not shown). Such a gender influence on immune response has been observed in humans (13–15). Although, it is recommended to use both male and female mice in pre-clinical studies to better represent the real-world scenario, we opted for female mice to improve the consistency of immune responses within groups (8). However, specific immune responses vary among different mouse strains, emphasizing the importance of evaluating different strains to identify the optimal immunogenicity model (16, 17). Both inbred and outbred strains have their advantages and disadvantages (18). Here, we assessed three inbred and one outbred mouse strains to identify the strain that elicited both total IgG and bactericidal antibody responses consistently to all four MenACYW conjugate vaccine serogroups. Among the mouse strains assessed, both inbred C3H/HeN and outbred ICR mice had a more dose-dependent antibody response to all the four serogroups than BALB/c and C57Bl/6 mice. In general, ICR mice had the greatest antibody dose-response (both total IgG and bactericidal) to all four serogroups. Moreover, the outbred mice better mimic the genetic diversity of humans than the inbred mice and are preferred for assessing candidate vaccines (18). Among the doses evaluated, the 0.25 µg per serogroup dose was chosen as optimal since this was in the dynamic range of the serogroup-specific dose-response curve in the selected ICR mouse strain; it elicited antibody responses (total IgG and bactericidal) to all four polysaccharides that were considerably different between those with the highest and lowest doses assessed. The dynamic range of the dose-response curve is expected to be the most sensitive to changes in formulation potency.

We showed that the three-dose schedule (prime and two boosters) elicited significantly higher antibody responses (total IgG and bactericidal) than the two-dose schedule (prime and a booster). However, we opted for the latter regimen to expedite the pre-clinical assessment time and to avoid potentially ‘saturating’ the antibody responses. With the three-dose schedule, delaying the bleed time by one week (from two to three weeks) after the last immunization resulted in a consistent decrease of total IgG responses for all serogroups, suggesting that the responses may have plateaued and thus not ideal for assessing potential changes in vaccine potency. Whereas, with the two-dose schedule the total IgG either stayed the same or slightly increased in serum samples obtained three versus two weeks after the last immunization. In addition, antibody responses following SC and IP administration were generally similar using the two-dose schedule. We opted for the SC route for our assessments going forward to minimize discomfort to animals; the IP route causes more severe pathological changes and discomfort to the animals than the SC route and hence the latter is recommended over the IP route (19).

Mice, like human infants (20), elicited a very weak or no antibody response to MenACYW (free polysaccharide) compared with the robust response to MenACYW-DT (conjugate vaccine) against all four serogroups. This may suggest that the mechanism of immune response is similar in naïve individuals, humans or mice. Bacterial polysaccharides being T-independent antigens are expected to elicit a poor antibody response in immunologically naïve populations. It was not unexpected to observe no to minimal, and predominantly of IgM class, antibody response in laboratory-bred mice that were never exposed to specific pathogens, including meningococcal bacteria, even after two immunizations with unconjugated polysaccharides vaccines (6). The optimized mouse immunogenicity model was also able to detect deterioration of the conjugate vaccine; total IgG responses decreased with the deteriorated conjugate vaccine, with reductions greatest for serogroup A followed by serogroups W and C, but with no effect on serogroup Y. These observations corroborate those by Beresford and co-worker’s (2017) who showed that serogroup A was the most sensitive to deterioration at elevated temperatures, resulting in complete depolymerization at 56°C (21). The outbred strain, ICR selected for the immunogenicity evaluation may not be as close as ‘dirty mice’ to humans as the latter typically harbor natural mouse pathogens, and therefore, mimic humans in the real world (22). Nonetheless, we believe that the identified pre-clinical immunogenicity model is well suited for the purpose of meningococcal vaccine development, because of the following reasons namely: (1) experimental reproducibility, (2) the observed immune response pattern in ICR mice is comparable to naïve infants, and (3) this mouse model was successfully used in the development and licensure of the meningococcal conjugate vaccine, MenQuadfi^®^ (23, 24).

There are biochemical techniques that can detect deterioration of the vaccine at its intended storage temperature. For example, measuring free polysaccharide levels would be one such technique ideal for the stability monitoring of polysaccharide-protein conjugate vaccines. Since the immunogenicity of polysaccharides depends on their covalent attachment to a carrier protein, the potency of such vaccines can also be indirectly inferred from the level of unconjugated (i.e., free) polysaccharides (25, 26). There are many sensitive biochemical and immunochemical analytical methods developed to characterize various components of conjugate vaccines and to monitor their integrity/stability, and for routine release testing (25). These methods examine different attributes of the polysaccharide-protein conjugates and the results are used alone or in combination to infer vaccine potency. Whereas, an optimized pre-clinical mouse immunogenicity model is a valuable tool that demonstrates the comprehensive potency of meningococcal conjugate vaccines. Pre-clinical immunogenicity is also used as one of the key critical quality attributes to demonstrate manufacturing consistency (26). However, a pre-clinical immunogenicity model is generally not ideal for routine release testing or stability monitoring of polysaccharide conjugate vaccines due to the inherent variability in the immune response between animals, the time required to complete assessments, and distress caused to the animals.

In conclusion, the ICR mouse strain immunized subcutaneously with 0.25 µg per serogroup polysaccharide-protein conjugate vaccine candidates on days 0 and 14, with serum samples obtained on day 28 for immunogenicity evaluation, is the best pre-clinical immunogenicity model to demonstrate proof-of-concept when assessing candidate meningococcal conjugate vaccines. We used this model to efficiently screen various conjugation chemistry-related parameters during early development of a meningococcal conjugate vaccine so as to select the most immunogenic formulations for progression to clinical assessment (9). This immunogenicity model has helped to stratify vaccine candidates for clinical studies and thus expedited our vaccine development and saved resources by limiting the number of lead formulations for clinical evaluation.

## Data Availability Statement

The original contributions presented in the study are included in the article/supplementary material. Further inquiries can be directed to the corresponding author.

## Ethics Statement

The animal study was reviewed and approved by Institutional Animal Care and Use Committee (IACUC), Sanofi Pasteur, 1 Discovery Drive, Swiftwater, PA 18370, USA. Written informed consent for participation was not obtained from the owners because Not applicable.

## Author Contributions

AA was responsible for conception and design of the studies, analysis and interpretation of data, drafting and critically reviewing the manuscript. AR and SV were responsible for conducting different animal studies and acquisition, analysis and interpretation of data, and critically reviewing the manuscript. All authors contributed to the article and approved the submitted version.

## Funding

This research was funded and sponsored by Sanofi Pasteur. All authors had full access to all the data in the study and had final responsibility for the decision to submit for publication.

## Conflict of Interest

AA and SV are employees of Sanofi Pasteur and may hold shares and/or stock options in the company. AR was an employee of Sanofi Pasteur at the time the study was conducted. The authors declare that this study received funding from Sanofi Pasteur. The funder had the following involvement with the study: study design, data collection, data analysis, data interpretation, writing of the report, and the decision to submit the paper for publication.

## Publisher’s Note

All claims expressed in this article are solely those of the authors and do not necessarily represent those of their affiliated organizations, or those of the publisher, the editors and the reviewers. Any product that may be evaluated in this article, or claim that may be made by its manufacturer, is not guaranteed or endorsed by the publisher.
